# Enzymatically-induced dynamic assemblies from surface functional stomatocyte nanoreactors†

**DOI:** 10.1039/d4tb01320d

**Published:** 2024-10-07

**Authors:** Alexander D. Fusi, Yudong Li, Marrit M. E. Tholen, Marlo Cieraad, Lorenzo Albertazzi, Tania Patiño Padial, Jan C. M. van Hest, Loai K. E. A. Abdelmohsen

**Affiliations:** a Faculty of Chemical Engineering and Chemistry, Eindhoven University of Technology Eindhoven The Netherlands t.patino.padial@tue.nl j.c.m.v.hest@tue.nl l.k.e.a.abdelmohsen@tue.nl; b Faculty of Biomedical Engineering, Eindhoven University of Technology Eindhoven The Netherlands

## Abstract

Collective behavior has become a recent topic of investigation in systems chemistry. In pursuing this phenomenon, we present polymersome stomatocytes loaded with the enzyme urease, which show basic stigmergy-based communication and are capable of signal production, reception, and response by clustering with surface complementary binding partners. The collective behavior is transient and based on the widely known pH-sensitive non-covalent interactions between nitrilotriacetic acid (NTA) and histidine (His) moieties attached to the surface of urease-loaded and empty stomacytes, respectively. Upon the addition of the substrate urea, the urease stomatocytes are able to increase the environmental pH, allowing the NTA units to interact with the surface histidines on the complementary species, triggering the formation of transient clusters. The stomatocytes display a maximum clustering interaction at a pH between 6.3 and 7.3, and interparticle repulsive behavior outside this range. This leads to oscillating behavior, as the aggregates disassemble when the pH increases due to high local urease activity. After bulk pH conditions are restored, clustering can take place again. Within the detectable region of dynamic light scattering, individual stomatocytes can aggregate to agglomerates with 10 times their volume. Understanding and designing population behavior of active colloids can facilitate the execution of cooperative tasks, which are not feasible for individual colloids.

## Abstract 2

Pursuing collective behaviors, we present enzyme-loaded polymersome stomatocytes with stigmergy-based communication capable of signal production, reception, and response by transiently clustering with surface complementary artificial species.

## Graphical abstract

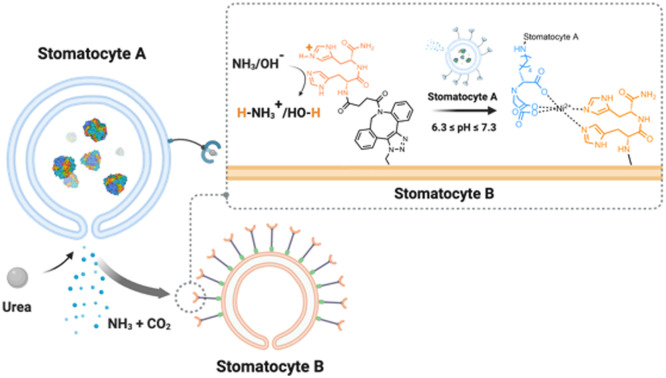

## 1. Introduction

Microorganisms are able to respond to population density through a series of complex phenomena, ranging from population-based signaling networks such as quorum sensing to predation and motility.^1^ One specific form of such collective behavior is described by the indirect communication model of stigmergy that leads to the emergence of spontaneous, coordinated conduct observed both at the macro level and at microscales in bacterial collective migrations or self-organization.^2–5^ Simply, the action of an agent induces an effect or “signal” in the surrounding medium of a population; this signal leads to the activation of a secondary agent, which triggers a coordinated action. As a result, without communicating directly with each other, individual microorganisms can display collective behavior mediated by the surrounding medium.^5^ Interestingly, stigmergic interactions lead to a series of benefits and system control optimizations, where no planning, memories of previous activity, mutual “awareness,” and individual commitment to an agent are necessary to complete a task. With a perceivable trace left in the medium, individuals can act according to the signal, coordinated task executions occur, and swarm “intelligence” rises. This type of behavior has been identified across several bacterial species for biofilm development and cell migration, *e.g.*, *Myxococcus xanthus* and *Streptococcus pyogenes*, where the dependency on unintentional environmental cues such as pH has been established.^6–8^

This naturally occurring collective behavior has inspired scientists to design synthetic analogs, which has proven to be far from trivial; challenges include platform design complexity and synthesis, physicochemical characterization, versatility, and robustness – all of which, to some extent, hinder the reach of collective, life-like properties.^9–12^ So far, the most effective examples show clustering and swarming of non-autonomous colloids with electric fields, light, or magnetism-mediated inputs.^13–15^ While these are relevant examples for remote guidance and cooperative executions of tasks, these platforms often operate under surfactant-rich or organic/water co-solvent mixtures and are mediated by constant external manipulation.^16,17^ Alternately, a more biomimetic approach employs enzymatic activity, which can induce chemical gradients resulting in autonomous action. A few options are proposed by Hest and Walther, who developed enzyme/DNA-based signaling circuits driven by catalytic cascades or by activator molecules (assembly-inducing) and dormant deactivators (disassembly-inducing) that regulate the lifetimes of clusters.^18–21^ However, these options present several disadvantages in terms of time duration (5+ hours for assembly) and reversibility, and they require an additional stimulus or components, such as dormant deactivators, to return to the initial disassembled state.^22^ Nonetheless, the study of enzymatically induced collective behaviors is still in infancy. Accordingly, the fundamental rules must be investigated further to apply artificial bioactive colloids in the mimicry of population behavior.^1,2,12,22–24^ Furthermore, we still lack an understanding on how enzymatically powered micro/nanomotors interact and self-organize based on local microenvironmental changes and the quality of the ions present in the medium, *e.g.*, kosmotropic and chaotropic salts.

**Fig. 1:**
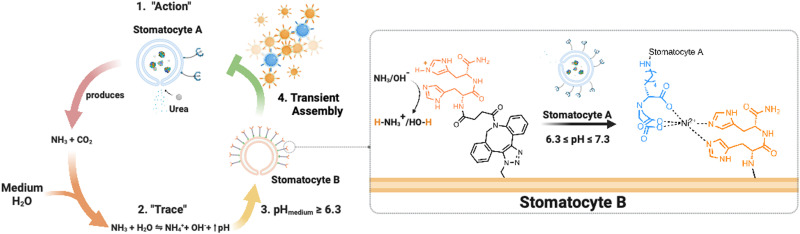
Overview of the stigmergy-based system design. 1. Production and release of ammonia (“action”) from urease-loaded, Ni^2+^–NTA–stomatocytes. 2. The product of the action interacts with the medium (surrounding solution) and increases the pH of the environment. 3. The basification of the environment leads to the indirect stimulation of His_2_-decorated stomatocytes by deprotonation of the surface histidines, enabling the pH-responsive NTA–Ni^2+^–His_2_ complex to form. Upon a further pH increase the assembly of multiple colloids disintegrates, making the process transient.

Here, we present an enzymatically powered, transiently clustering nanoreactor platform that operates with the essential features of stigmergy. For this purpose, we employ nitrilotriacetic acid (NTA)-functional, urease-loaded polymersome stomatocyte (urease-stomatocytes) nanoreactors (“agent A”), which convert urea into ammonia and carbon dioxide within their nanocavity. Upon the production and release of the ammonia “signal” from the urease-loaded population of stomatocytes, the pH of the medium is altered to basic conditions (“trace”), which leads to the deprotonation of the surface-bound histidine ligands on a second population of empty stomatocytes (“agent B”). This enables affinity tag-based, interparticle clustering and a stigmergy-based form of particle communication (Fig. 1). When the pH surrounding the urease-loaded stomatocytes overshoots due to the high local concentration of the urease nanoreactors, colloidal repulsive forces overtake the attractive NTA–Ni^2+^–His_2_ forces, and cluster disassembly occurs. Subsequently, the pH surrounding the nanoreactors equilibrates with the medium, which resets the system and allows for a second round of assembly.

The current work establishes a transient assembly/disassembly system. With this platform, we provide a baseline for an active colloidal system that operates in physiological conditions and outputs transient clustering autonomously without additional stimuli. Moreover, the study highlights the relevance of the “medium” composition in affecting clustering. It provides a foundation for more elegant biomimicking, signal-adaptive systems, and ways to mimic and understand natural biological behaviors. Lastly, the asymmetric polymersome stomatocyte morphology allows the coupling of enzymatically induced motion to the dynamicity of non-equilibrium self-assembly behaviors.

The establishment of this platform enables the development of systems that can shift from basic non-equilibrium self-assemblies to self-organization, an aspect that would elucidate the fundamental components of the emergence of life and is severely understudied with colloidal systems and enzymatic action.^12^

## 2. Experimental

### 2.1 Materials

Poly(ethylene glycol) methyl ether (*M*_n_ = 2 kDa; Sigma-Aldrich, 99%), α-hydroxy-ω-azido PEG (*M*_n_ = 3 kDa; Rapp Polymere), α-bromoisobutyryl bromide (Sigma-Aldrich, 98%), triethylamine (Merck, 99%), styrene (Reagentplus® Sigma-Aldrich, ≥99%) *N*,*N*,*N*′,*N*′′,*N*′′-pentamethyl-diethylenetriamine (PMDETA) (TCI, ≥99%), neutral alumina oxide (activated, Brockmann Activity I) (Sigma-Aldrich), dibenzocyclooctyne-acid (DBCO-acid) (TCI, >98%), Fmoc-His(Trt)-OH (Novabiochem®), Sieber amide resin (100–200 mesh, loading 0.40–0.80 mmol g^−1^, Novabiochem®), *o*-(benzotriazol-1-yl)-*N*,*N*, *N*′,*N*′-tetramethyluronium hexafluorophosphate (HBTU) (Biosolve, 98%), diisopropylethylamine (DIPEA) (CARLO ERBA Reagents, peptide grade), piperidine (CARLO ERBA Reagents, peptide grade), trifluoroacetic acid (TFA) (CARLO ERBA Reagents, peptide grade), dichloromethane (Biosolve), dimethylformamide (Biosolve), AZDye 405 DBCO (Click Chemistry Tools), AZDye 647 DBCO (Click Chemistry Tools), AZDye 488 DBCO (Click Chemistry Tools), SNARF™-4F 5-(and-6)-carboxylic acid (Thermo Fisher, 96%) NTA-PEG-DBCO (PEG-*M*_n_ = 3.4 kDa, Nanocs), urease from *Canavalia ensiformis* (Type IX, 72.5 U mg^−1^), and urea (Sigma-Aldrich, ≥98%) were used as received. The azido-terminated macroinitiator for atom transfer radical polymerization (ATRP) and copper(i) bromide (Cu(i)Br) (Sigma-Aldrich, 98%) were purified according to previously reported procedures.^22^ All solvents used were peptide synthesis grade.

### 2.2 Equipment

#### Cryogenic transmission electron microscopy (Cryo-TEM)

Experiments were performed on the TU/e CryoTitan (Thermo Fisher Scientific) equipped with a field emission gun and autoloader and operated at 300 kV acceleration voltage in low-dose bright-field TEM mode. Samples for cryo-TEM were prepared by glow-discharging the grids (Quantifoil Cu grid with R 2/2 holey carbon films, Quantifoil Micro Tools GmbH, part of the SPT Life Sciences group) in a Cressington 208 carbon coater for 40 seconds. Then, 3 μL of samples were pipetted on the grid and blotted in a Vitrobot MARK IV at room temperature and 100% humidity. The grid was blotted for 3 seconds (offset −3) and directly plunged and vitrified in liquid ethane. Cryo-TEM images were acquired with zero loss energy filtering mode (Gatan GIF 2002, 20 eV energy slit) on a CCD camera (Gatan model 794). All electron microscope images were processed with Fiji 2.7.0 software (ImageJ2).

#### Dynamic light scattering (DLS) and zeta potential (ζ)

A Malvern Zetasizer Nano ZSP equipped with a 633 nm He–Ne laser was used to record the hydrodynamic size, surface zeta potential, and clustering behavior of the particles at room temperature.

#### Fluorescence microscopy

Fluorescence images were obtained on an Oxford Nano Imager equipped with a 100 × 1.4 NA oil immersion Olympus objective and an ORCA Flash 4 sCMOS (Hamamatsu) camera.

#### Size exclusion chromatography (SEC)

The molecular weights and distributions of the synthesized polymers were determined through a Shimadzu Prominence-I SEC system with a PL gel 5 μm mixed D and mixed C column (Polymer Laboratories) calibrated with PS standards and equipped with a Shimadzu RID-20A differential refractive index detector. The eluent THF was used with a flow rate of 1 mL min^−1^.

### Liquid chromatography-mass spectrometry (LC-MS)

The synthesized dibenzocyclooctyne-histidine dimer was analyzed on an LC-MS system (PDA detector and Thermo Scientific LCQ Fleet Ion Trap Mass Spectrometer) coupled to a high-performance liquid chromatography pump with Phenomenex Kinetex 2.6 μm EVO C18 50 × 2.1 mm column, by a linear gradient elution 5–100% of acetonitrile in MQ (0.01% formic acid).

#### Nanodrop protein analysis

Protein concentrations were determined by recording the absorbance at 280 nm on a Nanodrop ND-1000 Spectrophotometer.

#### NanoSight tracking analysis

Particle concentrations and tracking analyses were obtained through a Malvern NanoSight NS300 instrument equipped with an sCMOS camera and a Blue488 nm laser diode at a rate of 25 frames per second.

#### Nanoparticle size exclusion chromatography

Urease stomatocyte nanoreactors were purified from free protein with a Superose™ 6 column equipped with a Shimadzu Prominence I SEC system. The eluent used was 1X PBS at a flow rate of 0.9 mL min^−1^.

#### Nuclear magnetic resonance spectroscopy (NMR)

^1^H-NMR spectra were obtained with a Bruker (400 MHz) spectrometer using CDCl_3_ as solvent (TMS internal standard).

#### Single particle automated Raman trapping analysis (SPARTA®)

SPARTA measurements were performed in collaboration with SPARTA Biodiscovery Ltd (London, UK) and performed on the SPARTA Alpha system (Stevens Group, Imperial College London).^25,26^

#### Plate reader

All absorbance and fluorometric measurements were performed on a Tecan™ MC Spark 10M plate reader using Corning Falcon 96 Flat Transparent well plates for enzyme encapsulation efficiencies or Nunc 384 Flat Black well plates. Fluorescence intensity readouts were obtained by recording the emissions with a settle time of 500 ms.

### 2.3 Synthesis of methoxy and azide-terminated poly(ethylene glycol)-*b*-poly(styrene)

According to previously reported procedures, the methoxy and azide terminated polymers were synthesized by atom transfer radical polymerization.^22^ Briefly, the macroinitiator (1 mol eq.) and a volume of anisole (1/6 v/v of anisole/styrene) were added to a Schlenk tube. To this, 400 mol eq. of styrene (basic alumina purified; sparged with argon) and 3 mol eq. of Cu(i)Br were added under argon counterflow, and the resulting mixture was sparged with argon for one hour. Then, 3 mol eq. of PMDETA were added directly to the reaction mixture, the solution sparged for one hour, and then placed into a 90 °C oil bath to initiate the reaction. The progress of the reaction was followed by ^1^H-NMR spectroscopy. Upon reaching the desired length of the styrene block, the reaction vessel was cooled down in liquid nitrogen to stop the polymerization. The purity, molecular weights and distributions of the developed polymers were determined by NMR spectroscopy and size exclusion chromatography, respectively (ESI,† Fig. S1–S4). The ATRP process yielded PS with degrees of polymerization of approximately 215 and 225 for the methoxy-terminated polymer and its azido counterpart, respectively (ESI,† Fig. S1–S4). The SEC analyses indicated dispersity indices (*Đ*) of 1.14 (mPEG-*b*-PS) and 1.24 (N_3_-PEG-*b*-PS) (ESI,† Fig. S4).

### 2.4 Synthesis and analysis of dibenzocyclooctyne-functional histidine dimer

The dibenzocyclooctyne-His_2_ peptide was synthesized using standard Fmoc solid phase peptide synthesis with a Sieber amide resin solid support. Each residue was coupled with 8 eq. (relative to resin loading capacity) Fmoc-protected amino acids, 16 eq. of HBTU, and 16 eq. of DIPEA in a DMF solution for 30 minutes. Prior to any coupling, Fmoc protecting groups were removed using 20% piperidine/DMF (v/v) for 15 minutes. After each coupling and deprotection step, the resin was washed four times with 4 mL of DMF. Lastly, the histidine peptide was capped with dibenzocyclooctyne-acid (1.5 eq.) with 2 eq. of HBTU and DIPEA, washed with four volumes of DMF and DCM each, and dried under vacuum overnight. Peptide cleavage and side-group deprotection while retaining the DBCO functionality of the expected peptide were performed according to previously reported literature by reacting the dried resin with 30 v/v% TFA/DCM for 2 hours.^27^ The filtrate was first diluted with dioxane (*V*_f_ = 50 v/v%), sequentially concentrated to a viscous solution *via* rotary evaporation, filtered through a 0.2 μm PTFE filter, and lyophilized. The DBCO-peptide was separated and analyzed by LC-MS. DBCO-peptide exact mass = 578.24 *m*/*z*; observed = 579.17 *m*/*z* (ESI,† Fig. S5).

### 2.5 Assembly of empty and enzyme-loaded polymersome stomatocytes

Empty and loaded azide-functional stomatocytes were assembled according to a previously reported literature procedure.^22^ The synthesized polymers were mixed in dioxane at a 5 to 95 wt% ratio of azide-terminated polymer to the methoxy-terminated counterpart. The mixture was diluted with four parts of THF to obtain a final polymer concentration of 10 mg mL^−1^. A volume of 1 mL of the polymer/organic solvent mixture was stirred at room temperature, to which 1 mL of Milli-Q water (18.2 Ω) was added by a syringe pump at the rate of 1 mL h^−1^ to yield solvent-swollen vesicular structures. Then, the assembled formulation was dialyzed (12–14 kDa MWCO RC membrane, flat width 25 mm; Spectrapor®) against 5 mM NaCl with dialysis water refreshments at *t* = 1 h and 23 h to finally obtain rigid, “closed neck” stomatocytes. Stomatocytes with encapsulated enzymes were prepared similarly, except for the addition process and dialysis water content. In the solvent-shift process, 0.6 mL of MQ water was initially added at the same flow rate to ensure the formation of polymersome structures. Thereafter, 0.4 mL of a urease enzyme solution in MQ (2 mg mL^−1^ by Nanodrop analysis) was added to the organic solvent mixture at the same addition rate. The enzyme/stomatocyte mixture was then dialyzed against a higher salt concentration (100 mM NaCl) compared to empty stomatocytes. Following the self-assembly process, the enzyme-loaded stomatocytes were concentrated to a final volume of 1 mL *via* spin filtration, and the free enzyme was removed by size exclusion chromatography using a Superose™ 6 column. The purified enzyme-loaded structures were concentrated over 0.22 μm PVDF low-binding Durapore® membranes, and the enzymatic loading efficiency was estimated by bicinchoninic acid (BCA) assays according to previously reported literature.^22^ Upon the analysis of enzyme encapsulation, a 3% incorporation efficiency was determined upon a 2 mg mL^−1^ feed. It is worth noting that some product was lost during the sample preparation for the BCA assay, as the enzyme needed to be released from the stomatocytes, thus potentially reducing the actual value of encapsulated efficiency to what was observed and reported.

### 2.6 Surface functionalization of polymersome stomatocytes

The stomatocyte suspension was concentrated by spin filtration over 0.22 μm PVDF filters to remove any structures with a diameter below 220 nm and resuspended in 1× phosphate buffer saline (1× PBS; pH = 7.4) to a final approximate polymer concentration of 0.5–1 mg mL^−1^. The suspensions were then aliquoted and surface-functionalized with DBCO-His_2_ or DBCO–PEG_67_–NTA in a two-step surface functionalization. The aqueous stomatocyte aliquot was mixed with either DBCO-ligand in DMSO (2 mg mL^−1^ stock concentration) to a final concentration of 15 : 1 molar equivalents of the ligand to the azide concentration in the suspension. The mixture was shaken at room temperature for a period of time (NTA samples *t*_reaction_ = 60 min; His_2_ samples *t*_reaction_ = 5 min). The suspension was then spun down and washed with 1X PBS five times to remove unreacted DBCO-ligands, resuspended to the original stomatocyte concentration with the same buffer, and mixed with 15 equivalents of a DBCO-functional fluorescent dye under the same conditions. The final suspension was then spun down and resuspended with 1X PBS buffer until no free dye was present in the solution. The sample was then compared to an aliquot that fully reacted with the same DBCO-fluorescent dye. The two aliquoted suspensions were diluted to similar particle concentrations of approximately 1 × 10^7^ particles mL^−1^ and analyzed for their fluorescence output by microplate reader assays. Under the assumption that the azide-terminated polymer distributes itself homogeneously across polymersome stomatocytes and between the inner and outer leaflet of the polymersome structure, we correlated the decrease of the fluorescence of the ligand-modified stomatocyte populations with a functionalization yield. The stomatocyte morphology retention post-click functionalization was determined by cryogenic transmission electron microscopy (ESI,† Fig. S6B). Empty and enzyme-loaded NTA-stomatocytes were charged using standard Ni^2+^–NTA bead resin charging solutions. The stomatocyte populations were shaken for 15 minutes in a 0.1 M NiSO_4_ solution at room temperature. Thereafter, the suspension was spun down over 0.1 μm PVDF filters and resuspended in more Ni·SO_4_ solution. The process was repeated three times, and the resulting nickel-charged NTA (Ni^2+^–NTA) stomatocytes were then spun down, resuspended with pH = 8 PB buffer, shaken for one minute, and spun down to remove any excess NiSO_4_. The washing step was repeated twice more, and the stomatocyte suspension was finally resuspended in a buffer at pH = 8 (5 mM HEPES, 10 mM NaCl, 10 mM imidazole). SPARTA measurements were performed in collaboration with SPARTA Biodiscovery Ltd (London, UK) on the SPARTA Alpha system (Stevens Group, Imperial College London).^25,26^ For each sample, 200 μL of particle suspension was placed on a 19 mm diameter cover glass (VWR) affixed to a microscope slide, and a 63 × 1.0 NA water immersion lens (W Plan Apochromat, Zeiss) was immersed in the droplet. Particle Raman spectra were obtained through 300 trapping acquisition attempts per sample; the acquisition time was set to 10 seconds per particle, and the laser disabling time was set to 2 seconds to allow the particles and larger aggregates to diffuse away from the confocal volume. The acquired data was then later processed with a spectral response correction (system calibration), cosmic spike removal, background subtraction (average of 20 spectra of background buffer 5 mM HEPES, 10 mM NaCl, and 10 mM imidazole measured at 10 s; ESI,† Fig. S7A), baseline correction, smoothing, and normalization under the curve. Raman spectra were preprocessed by adding a spectral response correction based on a 785 nm excitation standard (National Institute of Standards and Technology, US, SRM2241) and manually removing aggregates or empty traps (ESI,† Fig. S7B). Next, a background subtraction of 95% was performed using a blank buffer signal, followed by a Whittaker baseline correction. A Savitzky–Golay smoothing filter was then applied to each spectrum with order 1 and frame size 7. Each particle spectrum was normalized to the area under the curve. Mean spectra and standard deviation were calculated across one sample in Matlab R2020a and plotted in OriginPro 2020b.

### 2.7 Colloidal characterization

Hydrodynamic size, surface zeta potential, and clustering behavior of the particles were recorded at room temperature (sample volume_final_ = 1 mL) on a Malvern Zetasizer Nano Zs. All sample outputs were recorded as triplicate measurements (*t*_sub-run_ = 10 s; 13 sub-runs per measurement). Alternately, the size evolution of multi-colloidal assemblies was determined by five hours of measurements with ten sub-runs (*t*_sub-run_ = 10 s) per measurement.

### 2.8 Nanosight tracking analysis of urease-loaded stomatocytes

Motion tracks and diffusion data were obtained with a Nanosight NS300 (sCMOS camera; Blue488 nm laser type; frame rate per second − FPS = 25.0) by diluting the urease-loaded stomatocytes to a final concentration of 0.025 mg mL^−1^ in MQ and urea at varying concentrations. Upon mixing, the enzyme-loaded stomatocytes were immediately vortexed for 5 seconds, quickly injected into the sample cell of the NS300, and their tracks recorded for 30 seconds. To ensure reproducibility, each sample was measured three times. The Nanosight Tracking Analysis 3.2 software was used to record and track the motion trajectories of individual particles. Particles with a persistence of 20 frames minimum were included in the diffusion analysis.

### 2.9 Fluorescence imaging of colloidal clusters

Fluorescence microscopy was carried out with an Oxford Nano Imager under total internal reflection (TIR; illumination angle_deg_ = 53.50) conditions to capture particles on the glass slide. Samples were prepared at relevant pHs, loaded into Ibidi μ-Slide VI 0.5 Glass Bottom flow chambers, and left to adsorb for 10 min prior to imaging. Laser channels of 473 nm and 640 nm (exposure time = 50 ms) were used to excite the fluorophores attached to the surface of the stomatocytes and videos of 200 frames (FPS = 20) with a total acquisition time of 10 seconds.

### 2.10 pH evolution studies

The pH evolution of the medium was determined using a C-SNARF-4F dye dissolved in the buffered solution. The ratiometric dye was prepared according to a literature-reported procedure except for the buffer contents used (5 mM HEPES, 10 mM NaCl, 10 mM imidazole) (ESI,† Fig. S9).^28^

### 2.11 Urease activity assays

The production of ammonia was determined using the Urease Activity Assay Kit (MAK120, Sigma-Aldrich) based on the Berthelot method to determine the activity of urease-loaded stomatocyte nanoreactors. Briefly, in a clear 96-well flat-bottom transparent plate, Urease-stomatocytes were diluted to a final concentration of 0.05 mg mL^−1^ in 100 μL of a urea solution in 1X PBS, and the plate incubated at room temperature for 5 minutes before stopping the ureolytic process. After adding the kit reagents, the plate was incubated for 30 minutes away from light at 37 °C and then cooled to room temperature before recording the absorbance at 670 nm. The obtained value was correlated to a calibration curve of ammonium chloride.

### 2.12 Statistical analysis

Unless stated otherwise, the data presented is listed as the mean ± standard deviation (SD) of triplicate measurements. Values presented from SPARTA Biodiscovery Ltd were calculated by the company in Matlab R2020a. C-SNARF 4F emission ratio curves were fitted through a nonlinear regression (fifth-order polynomial). All statistical results were performed using Prism 10.0.1 software (GraphPad Software, inc., USA).

## 3 Results and discussion

### 3.1 Fabrication, surface functionalization, and characterization of stomatocyte nanoreactors

**Fig. 2:**
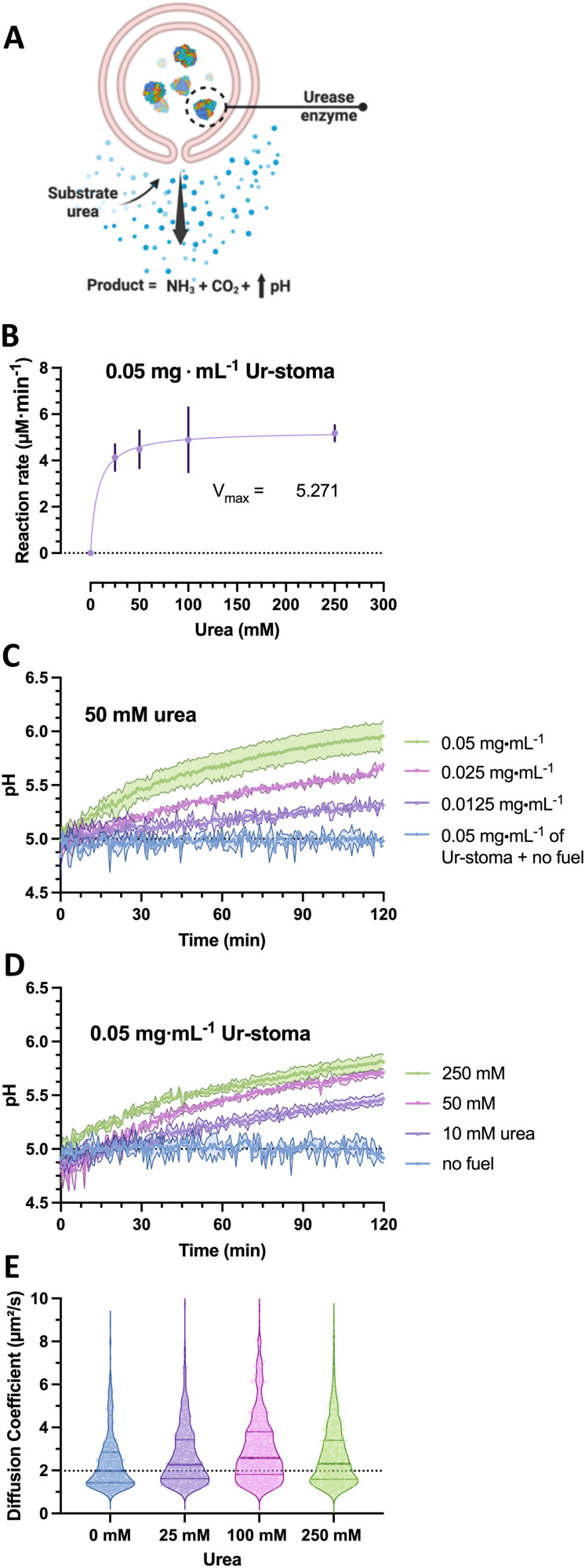
(A) Overview of urease-stomatocyte catalysis and motion. (B) Urease (type IX) loaded polymersome stomatocyte conversion rates as function of urea substrate concentrations in assay kit buffer (pH = 7.0). (C) pH evolution as a function of nanoreactor concentration. (D) pH evolution in presence of 0.05 mg mL^−1^ urease-stomatocytes as a function of fuel concentration. (E) Diffusion coefficients of urease-stomatocytes as a function of urea concentration (*n* = 500 particles per sample type).

To construct the stigmergy system, two types of colloids need to be prepared: bowl-shaped polymersomes, or stomatocytes, filled with the enzyme urease and empty stomatocytes. To enable interaction between these colloids, they were decorated with either Ni–NTA ligands or histidine dipeptides. This made the particles susceptible to the action of the enzymatic stomatocyte nanoreactors, which converted urea into ammonia and CO_2_ (Fig. 2(A)), leading to a pH increase that was perceived by the histidine-decorated empty stomatocytes. This “trace” perception subsequently led to inter-particle complexation and clustering.

To attain the stomatocytes, methoxy- and azido-terminated poly(ethylene glycol)-*b*-poly(styrene) (m-/N_3_-PEG-*b*-PS) copolymers were synthesized by ATRP using methoxy/azido-PEG_44/67_-based macroinitiators and styrene to yield macromolecules with well-defined blocks, physicochemical characteristics, and an exposed functional handle for post-assembly surface functionalization *via* click chemistry. Post-synthesis, the polymers were assembled into solvent-swollen vesicular structures through a gradual solvent switch from an organic solvent mixture to water; the presence of organic solvents allows vesicle bilayer flexibility and high sensitivity to osmotic pressure. By dialyzing these solvent-swollen structures in solutions of sodium chloride, a quick outflow of solvents from the structures can be achieved and induces an osmotic shock, which leads to bilayer bending and volume reductions into well-defined, bowl-shaped structures with azide moieties within and outside the lumen. These structures can be either assembled without enzymes or alongside urease type IX from *Canavalia ensiformis* in one pot (3% incorporation efficiency upon a 2 mg mL^−1^ feed during assembly), according to previously reported protocols.^22^

In the case of the nanoreactor, the ability of urease-loaded stomatocytes to produce ammonia and alter the pH were evaluated by enzyme activity assays and by analyzing the pH evolution from a low pH level. Here, the kinetics of the urease-loaded nanoreactors were determined by the Berthelot method, and the catalytic activities at the 5-minute mark compared as a function of urea substrate concentration. At a fixed concentration of 0.05 mg mL^−1^ of urease stomatocytes, the enzymes reached their maximum conversion rate at concentrations above 100 mM urea of approximately 5.2 μM ammonia min^−1^ (Fig. 2(B)). The plotted conversion rate curve aligned with the enzyme kinetic profiles of previously reported studies, with values above 50 mM of urea reaching the maximum conversion rate of the stomatocytes.^29^ In light of the quality of the enzyme received, the stochasticity of the enzyme encapsulation process, the loaded enzymatic content, and nanoreactor concentrations, the values reported here are in the comparable range of catalytic enzymatic activities presented in the works of Sanchez *et al.* and of our group with similar structures or the same used enzyme.^22,23,29,30^

Following the enzymatic activity assays, the ability of the urease-stomatocytes to alter the pH of the medium by the production of ammonia was further evaluated in buffer (5 mM HEPES, 10 mM NaCl, 10 mM imidazole) to determine the operating time. The buffer combination was selected as the least aggregation-inducing set of components for each species and its buffering range of 6.2–8.6. In addition, the use of imidazole prevented aspecific interaction, kinetic trapping, and irreversible aggregation.

Significant changes in the overall pH change during two hours of catalytic activity were observed as a function of stomatocyte nanoreactor or urea fuel concentration. With increasing concentrations of urease-stomatocytes from 0.0125 to 0.05 mg mL^−1^, the pH change obtained shifted from 0.3 to approximately 1 unit within two hours (Fig. 2(C)), whereas increasing fuel concentrations from 10 mM to 250 mM enabled greater reaction rates (Fig. 2(B)–(D)). Increasing the fuel concentration above the saturation point yielded, as expected, minor pH differences over two hours.

Given that urease is a well-known enzymatic chassis in the field of micro-/nanomotors, we also tested the colloidal nanoreactors for their capacity to move. Indeed, increased diffusivity was observed as the average diffusion coefficient shifted from a median of 1.97 μm^2^ s^−1^ up to 2.28–2.54 μm^2^ s^−1^ in the presence of fuel concentrations between 25 and 250 mM urea (Fig. 2(E)); however, due to the little difference in diffusivity and low encapsulation, potential motile behaviors of the stomatocytes can be ignored. Overall, the enzyme is still active following the nanoreactor assembly and organic solvent exposure, and pH evolutions can be steered by varying substrate concentrations and reactors. All consequent work was conducted with an enzymatic feed of 5 mg mL^−1^ (by Nanodrop analysis) during the assembly process.

Regarding the surface functionalization of the assembled structures for trace perception and coordinated clustering, the stomatocytes were assembled with a percentage of azide-terminated polymers and then functionalized with a combination of either DBCO–PEG_67_–NTA or DBCO-His_2_ with a fluorescent dye by strain-promoted azide–alkyne cycloaddition.

The surface functionalization was assessed with single particle automated Raman trapping analysis (SPARTA), a fluorescence spectroscopy method, and zeta potential measurements. Due to the difficulty in determining the azide polymer homogeneity in distribution throughout the formulation and in detecting the typical disappearing chromophore of DBCO at 310 nm in the PEG–PS system, these additional techniques were employed to corroborate the successful co-assembly and click reaction on the surface of the particles.

**Fig. 3:**
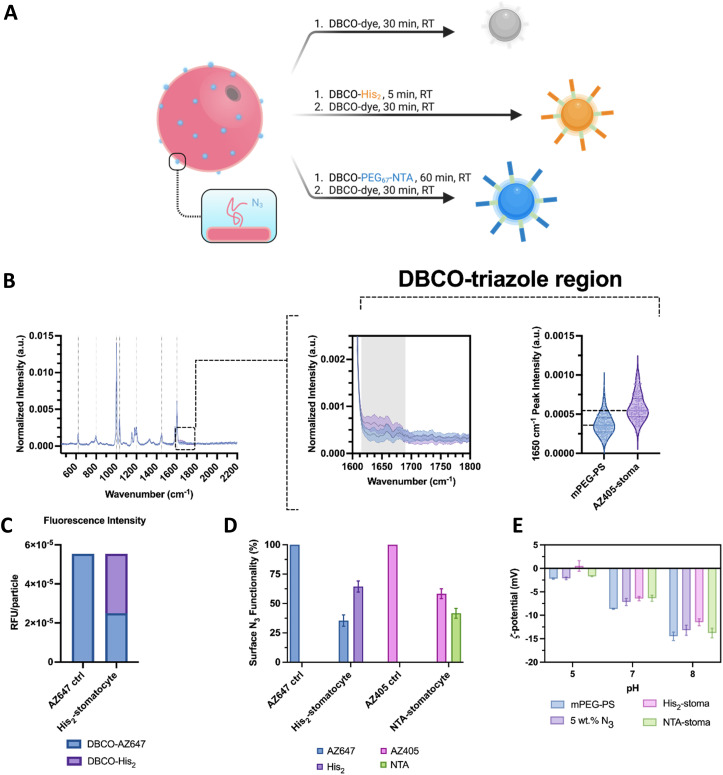
(A) Graphical overview of the two-step surface functionalization of stomatocytes by copper-free click reactions. (B) SPARTA analysis of stomatocytes (left-to-right) Raman spectrum overlay of methoxy-terminated and dye-functionalized PEG–PS stomatocytes, mean Raman spectrum crop of the aromatic double-bonded region corresponding to the triazole and conjugated region, and violin plots of peak intensity of the triazole region at 1650 cm^−1^ (*n* > 180 particles per sample; center line, median; upper and lower line, interquartile range). (C) Relative fluorescence intensities per particle of a stomatocyte sample fully reacted with the dye and one co-functionalized with histidine dimers. (D) Surface N_3_ occupancy based on the difference of the bulk fluorescence intensity output between stomatocytes fully conjugated with a dye and co-functionalized with a pH responsive moiety. (E) Zeta potential measurements of the stomatocyte sample types at different pHs in 5 mM HEPES, 10 mM NaCl, imidazole.

In the first type of analyses, a sample assembly formulation with 5 wt% azide terminated polymer was reacted with a fluorescent dibenzocyclooctyne-dye, the excess dye was removed, and particles from the assembly were analyzed further by Raman and fluorescence spectroscopy. Analysis by SPARTA showed no peaks of unreacted azides in the spectral region around 2100 cm^−1^ (Fig. 3(B)).^25^ The obtained Raman mean spectra displayed significant poly(styrene) signals with strong peaks at 1002 cm^−1^ and 1603 cm^−1^, corresponding to the ring breathing and stretch modes. The low standard deviations in the mean spectra indicated a homogeneous composition between particles. Moreover, it was possible to see the presence of peaks and notably higher intensities at 1600–1750 cm^−1^ in the clicked sample compared to the bare stomatocytes, which is associated with the aromatic and double-bonded vibrations of the DBCO–triazole complex (Fig. 3(B)-center). Further SPARTA analyses were performed on samples with NTA groups for their nickel charging capacity. Here, several peaks with increased peak intensity were observed, *e.g.*, at 1375 cm^−1^. Unfortunately, some of the peaks observed overlap with the strong PS signal of the stomatocyte body. Nonetheless, the peak at 580 cm^−1^ was investigated further as it was distinct from PS and was reported to be associated with NTA in previous literature (ESI,† Fig. S10D).^31^ The average peak intensity of each charged replicate was higher than the neat stomatocytes, the dye-labeled ones, and the uncharged sample (ESI,† Fig. S10E). It is possible that nickel complexation would increase the intensity of the signals detected due to the possible NTA polarization increase with the complexation of a metal ion. Nonetheless, the repeatability across particles and replicates suggests a detectable presence of nickel and the successful charge of the surface NTA groups.

To understand the ratio between NTA or His_2_ ligands and fluorescent dyes on the surface of stomatocytes, fluorescence intensity measurements were performed by plate reader, comparing fully conjugated, fluorescent dye-labeled stomatocytes and co-functionalized species. In modifying the surface of stomatocytes, aliquots of assembled structures were functionalized with either NTA–PEG_67_–DBCO and AZ405/AF488 or the complementary DBCO pair His_2_ and AZ647 and compared to a stomatocyte aliquot fully reacted with the associated fluorescent dye. By diluting the samples to the same particle number, the reduced fluorescence in the co-functional sample was an indication that a fraction of the azides had reacted with the binding ligands, which led to an estimation of the degree of functionalization (NTA or histidine dimer, Fig. 3(C) and (D)). Assuming homogeneous localization of the azide-terminated polymers across both bilayer leaflets and the Raman data, both stomatocyte layers react with DBCO molecules. However, the reacted decorations within the lumen of the stomatocyte are not accessible to other colloids and, therefore, are not considered critical in assembling multiple colloids. The reaction conditions used yielded stomatocyte surface functionalizations of approximately 78% for the NTA samples and 60% for the His_2_–stomatocytes.

Lastly, the successful attachment of the pH-sensitive surface ligands was validated further by zeta potential measurements at different pHs in 5 mM HEPES, 10 mM NaCl, and imidazole, where the histidine-functionalized samples showed distinctly different values compared to the unfunctionalized and NTA-functional stomatocyte samples (Fig. 3(E)). Histidine samples showed zeta potentials of 0.5, −6.4, −11.5 mV at pH 5, 7, and 8 respectively. Alternately, the NTA-functional samples expectedly showed similar zeta potentials to the unfunctionalized samples, namely −1.7, −6.4, and −13.8 mV and pH 5, 7, and 8. Combined with the lack of azide-associated peaks at 2100 cm^−1^ in the Raman analyses, this indicates that the click reaction was successfully performed. Regarding functionalization ratios, the conditions used were dependent on the aggregation behavior of each species on its own in 5 mM HEPES, 10 mM NaCl, and imidazole. The stomatocyte nanoreactor species at all percentages of NTA decorations were colloidally stable and did not flocculate nor sediment out of the solution. Alternately, histidine functionalizations up until 95% were possible; however, a high degree of his_2_ functionalizations led to severe colloidal aggregation and flocculation due to the chemical nature of the dimer. Lower concentrations of histidine moieties help in colloidal stability and reduced aspecific interactions. Nevertheless, concentrations below 40% his_2_ functionality were not enough to overtake the electrical double-layer forces of the colloids and enable any interaction. Therefore, 80% NTA functionalization was kept for the nanoreactors, whereas 60% was used for the histidine.

### 3.2 Colloidal forces at play

In order to understand the general clustering behavior as a function of pH without enzymatic action, empty nickel-charged nitrilotriacetic acid–stomatocytes and the histidine–stomatocytes were mixed to a final 1 : 4 population ratio in MQ water at pH = 4. The samples were titrated to higher pHs to determine the interaction of stomatocytes as a function of pH, and the static clustering ability was evaluated by dynamic light scattering (DLS) as a function of the cluster size at a specific pH point. Here, no significant size differences were visible across different pHs (ESI,† Fig. S11A). Examining the correlation functions revealed a distinct secondary decay after 1000 μs at the approximate pH = 7.2, generally associated with larger colloids in solution (ESI,† Fig. S11B and C). Unexpectedly, increasing the pH to 8 reduced the secondary peak to lower correlation values, indicating the disappearance of these larger species. Given the pH dependency and nature of the strong coordination bond between Ni^2+^–NTA and His_2_, it was expected to observe particle clusters and larger sizes at any pH above the p*K*_a_ of the histidine side chain.

**Fig. 4:**
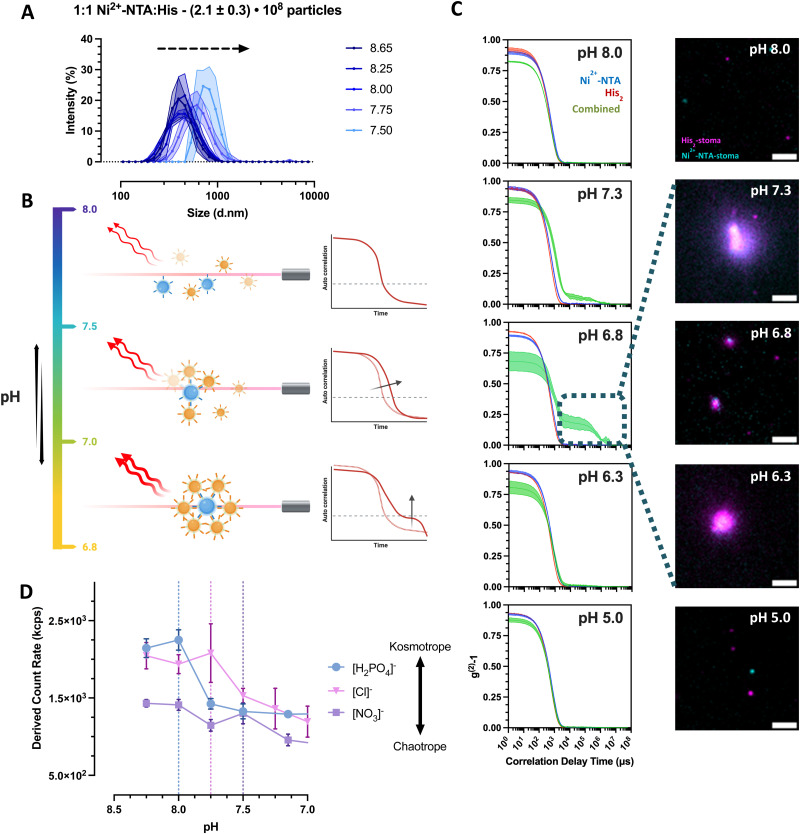
Clustering behavior of empty Ni^2+^–NTA– and histidine stomatocytes populations mixed at a 1 to 1 ratio in 5 mM HEPES, 10 mM NaCl, imidazole titrated to different pH levels. (A) Recorded hydrodynamic diameters of stomatocytes and clusters as a function of pH. (B) Exemplifying graphic for the observed correlation functions of the titrated stomatocyte samples. (C) (Left-row) DLS correlation functions of the individual stomatocyte types and combinations at different pH levels (blue curve = Ni^2+^–NTA–stomatocytes; red = histidine stomatocytes; green = combined 1 : 1 sample). Exemplary fluorescence microscopy images of aggregated and sedimented Ni^2+^–NTA– (AF647; cyan color) and histidine stomatocytes (AF488; magenta color) populations mixed at a 1 to 1 ratio at different pHs (scale bar = 2 μm; images altered for clarity purposes due to the difference in labeling across stomatocyte type; representative microscope images and area crops are available in ESI,† Fig. S12). (D) Empty Ni^2+^–NTA– and histidine stomatocytes populations mixed at a 1 to 1 ratio in 5 mM HEPES, 10 mM NaCl, imidazole titrated to different pH levels using phosphoric acid or nitric acid (vertical dotted lines = highlighted pH level after which a significant drop in the derived count rate can be observed).

To examine the observed phenomena further, various diluted buffers commonly used with Ni–NTA protein purification beads were evaluated to screen the repulsive forces of the stomatocytes and buffer the clustering pH range to elucidate the behavior of the complementary colloids. Unless stated otherwise, all experiments were done with a final population ratio of 1 : 1 Ni^2+^–NTA : His_2_ (*V*_f_ = 1 mL) and in 5 mM HEPES, 10 mM NaCl and imidazole. In this medium, the stomatocytes displayed maximal interaction only at pHs approximately between 6.5 and 7.5, with no relevant interactions at higher or lower pHs. By titrating from pH = 8 to 5, it was possible to see an increase in the intensity average population size alongside the emergence of a more distinct secondary decay corresponding to larger species in the suspension (Fig. 4(A)–(C)). The size change was detectable until pH = 7.5 in colloidal species up until 1 micron in diameter, corresponding to clusters of 8 partners minimum (Fig. 4(A)). Hereafter, size analyses of the assembled systems were not feasible due to the accentuated sedimentation of larger structures. The sedimentation of large clusters was supported further by the drop in the associated count rate and confirmed visually by TIRF microscopy (Fig. 4(C)).

To explain the lack of aggregation at pH = 8, we additionally sought to investigate the effects of counterions and electrolytes by titration with different ions of the Hofmeister salt series. Herewith, it was possible to modulate the onset of the cluster formation with the addition of kosmotropic (pH_onset_ ∼ 8) *versus* chaotropic (pH_onset_ ∼ 7.5) anions (Fig. 4(D)). The higher polarity of the kosmotropic phosphoric acid and its ability to form hydrogen bonds induce the preferential exclusion of the resulting anion from the ionic layer of the colloids, thus reducing the solubility and increasing the aggregation of the predominantly nonionic, PEGylated surface of the stomatocytes.^32,33^ The titration of the less kosmotropic hydrochloric acid and chaotropic nitric acid is believed to cause an energetically unfavorable disruption of the water structure, and the resulting anions are more associated with the electrical double layer (EDL) of the stomatocytes. The larger solvent-accessible, colloidal surface area augments its hydrophilicity and solubility, destabilizing the aggregates in the suspension. The critical clustering point was shifted closer to the stage where the EDL forces were superseded by the NTA–Ni^2+^–His_2_ complexation attractive forces.

Zeta potential measurements of the individual species at different pHs elucidated further the stability ranges of the complementary pairs with lower instability at high pHs for surface qualities analyzed due to the more significant negative EDL of PEG and high aggregation forces at physiological and lower pHs (Fig. 4(E)). At physiological conditions, the combination of low colloidal stability and the favorable NTA–Ni^2+^–His_2_ complex formation induced the clustering of complementary colloids, with a maximum interaction at pH ∼ 6.8–7 depending on the electrolyte quality and content. Stability at more acidic pHs was assumed to result from the imidazole in the buffer, preventing premature associations due to the protonation of surface histidines that cannot form complexes with the complementary Ni^2+^–NTA. Higher ionic strength and lack of imidazole from the medium more effectively screened the repulsive forces across colloids (ESI,† Fig. S11D); they created uncontrollably large clusters that cannot be analyzed by the Zetasizer. However, we continued with the current electrolyte content to ensure more control of assembly sizes and transient behaviors.

### 3.3 Population dynamics

**Fig. 5:**
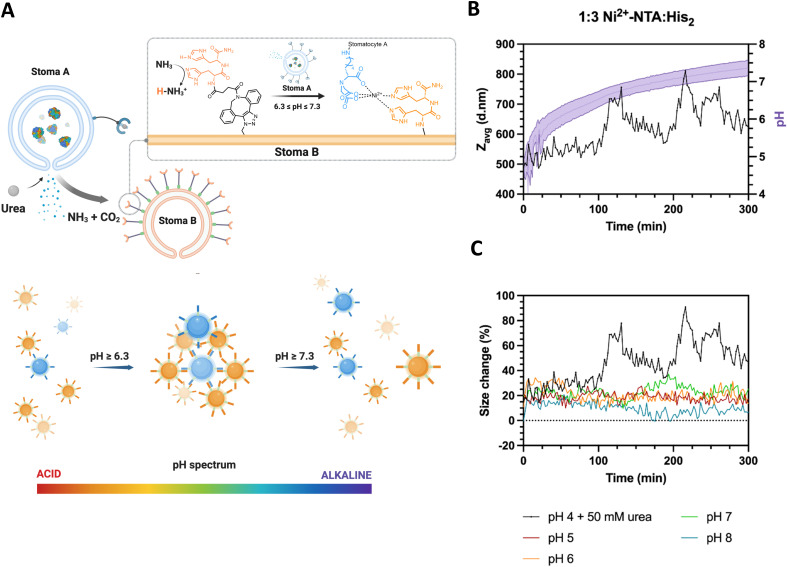
(A) Overview of system design of the autonomous production of ammonia from urease-loaded, Ni^2+^–NTA–stomatocytes (population A) and stigmergy based response of the empty, His_2_–stomatocytes, which respond to the increasing pH of the medium by having their surface ligands deprotonated, leading to colloid assembly. Nitrilotriacetic acid functional, urease-loaded stomatocytes catalyze the production of the signal ammonia, which leads to the deprotonation the surface of the surface complementary histidine-functional stomatocytes. Upon deprotonation, the histidines are able to form a complex with the Ni^2+^–NTA surface moieties of the complementary motors and colloidal clustering occurs. (B) pH evolution and *Z*_AVG_ of clustering stomatocytes measured over time (50 mM urea).(C) Normalized *Z*_AVG_ size increase of enzymatically induced clustering over time (0% = 400 d nm stomatocytes; 100% = 800 d nm); pH 5 to 8 lines represent.

To determine the stigmergic behavior of the particles, urease-loaded Ni^2+^–NTA–stomatocytes were mixed with their empty histidine counterparts at a 1 : 3 ratio in 5 mM HEPES, 10 mM NaCl and imidazole, and fuel was added to a final urea concentration of 50 mM (Fig. 5(A)). The pH and size evolution were monitored over five hours by fluorescence intensity with the pH-sensitive, ratiometric dye C-SNARF 4F and by DLS.

With the addition of fuel and after 100 minutes of incubation, we observed an oscillating pattern of average size increases and decreases from the typical stomatocyte hydrodynamic diameter of 400 d nm to 800 d nm and back, coinciding with the pH window that was determined as the clustering range of the colloids (Fig. 5(B) and (C)). In the pH evolution cycle, we propose that urease-loaded stomatocytes produce the signal ammonia, which alters the ambient conditions surrounding the colloids. Then, the ammonia trace persists in the environment, leading to a pH increase and consequent clustering of the colloids. Further local production of ammonia strengthened due to clustering shifts in the local pH of the cluster out of the complexation range, inducing an increased repulsion and disassembly from the increased electrical double layer of colloids. The local pH normalizes hereafter, and the clustering process is repeated until the bulk pH is outside the clustering range.

The fluctuating aggregated state of the colloids was also reflected in the fluctuations in diffusion coefficient within the samples as determined by the zetasizer. The cluster growth was accompanied by a decrease in the observed diffusion, which is inversely proportional to the particle/cluster diameter (ESI,† Fig. S13A). Furthermore, the lack of substantial decreases in the derived count rate over time indicated that clustering and dissociation occurred without any sedimentation when compared to samples mixed at different pH without fuel (ESI,† Fig. S13B).

Given the average stomatocyte hydrodynamic diameter of 400 nm, the average intensity size recorded in the first 100 minutes is still noticeably 20% larger than that of the individual colloids. With the inclusion of imidazole in the buffer preventing aspecific interactions from the NTA–Ni^2+^–His_2_ complex and the sensitivity of highly scattering large particles, we infer the presence of a small fraction of interacting particles in the medium, which are not capable of clustering beyond 20% due to the pH_bulk_ being outside the defined complexation range of the complementary colloids. It is hypothesized that the pH surrounding the colloid differs from the bulk pH due to the electrical double layer character and the production and diffusion of ammonia from the colloids to the remaining solution.^34–37^ During the evolution of the pH by the activity of the enzyme, we record the bulk pH change of the suspension and do not discriminate between the interfacial colloid pH and the rest of the solution.

**Fig. 6:**
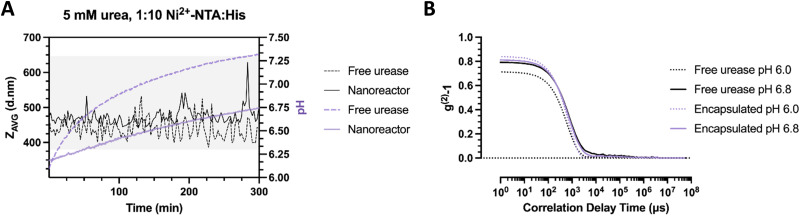
(A) pH evolution and *Z*_AVG_ of clustering stomatocytes measured over time (5 mM urea) with urease encapsulated in the nanoreactors or free (0.1 nM urease by Nanodrop analysis) in solution. (B) Associated correlation curves of clustering stomatocytes with free or encapsulated urease at pH 6.0 and 6.8.

To show the effect of local pH evolution more clearly, we compared samples (particle ratio – 1 : 10 Ni^2+^–NTA : His_2_) where the enzyme is encapsulated or free in solution. Here, the bulk pH is altered faster in the free urease sample compared to the nanoreactors. In free enzyme sample and at initial time points, DLS hydrodynamic diameter recordings begin at a size of 420 nm (Fig. 6(a)). Within the pH complexation range, the average size has been noted to increase up to 520 nm and to periodically orbit between 420 and 500 nm in diameter, which corresponds to a diameter increase of 20% compared to the original stomatocyte size. In the case of the nanoreactor, the average size of the first measurement was approximately 470 nm in diameter, displaying a considerable size increase with a slower pH evolution compared to the free enzyme. In addition, the clustering size range reached 630 nm, elucidating a substantial effect resulting from the encapsulation of the enzymatic chassis. Alternately, the difference in pH evolution speed and clustering size range indicates a diffusion-limited, constant local pH evolution around the nanoreactor that gradually induces local aggregation without reaching the overshoot pH and colloidal disassembly.

In contrast, the fast local pH change from the free enzyme may induce a strong overshoot, which would directly impede the time necessary for colloids to be controllably affected by the produced ammonia gradient and assemble. A closer inspection of the correlation data displays an increase in the average size for both samples from the delayed decay of the first peak at pH 6.8 compared to pH 6.0. However, the slope of the nanoreactors at pH 6.0 appears steeper compared to the free enzyme, which indicates a greater dispersity in the latter sample to begin with (Fig. 6(b)). The greater initial dispersity suggests protein adsorption to the surface of colloids, which may result in protein-covered affinity tags, less opportunity for complexation, and smaller cluster sizes achieved. The current setup suggests a time-dependent assembly formation. Nevertheless, additional confirmation by microscopy is necessary to confirm the adsorption of proteins to the surface of stomatocytes and local pH evolution dependency.

Given the current system, the stomatocytes can controllably assemble into smaller clusters up to 0.8 μm within two hours of urea addition. Larger clusters can be obtained by tailoring the species ratios, buffer quality and capacity of the medium, and fuel concentrations. A species ratio of 1 : 1 may enable an ensemble of diffusion and reaction-limited aggregations and larger-sized clusters observed in the works of Sen and Walther (1+ μm). In contrast, skewed proportions may cap the cluster size.^18,19,38^ Additionally, increasing the ionic strength and removing the imidazole from the medium may screen the repulsive forces across colloids, albeit at the cost of reversibility of interactions and the defined clustering range.^39^ Lastly, it is important to note the chaotropic nature of urea and its ability to stabilize colloids.^33^ Consequently, high fuel concentrations may prevent large colloidal co-assemblies. In contrast, large product concentration gradients may induce uncontrolled large aggregates, exemplifying the complexity of feedback-dependent platforms, systems chemistry, and biological interactions.

According to the results, fuel does not need to be added consistently throughout the experiment to enable oscillating behaviors. However, to cycle the complete pH evolution process, the buffer contents must be refreshed, supplemental urea added, and pH levels restored. First, a high ammonia concentration in the solution would shift the pH to levels where the complex between stomatocytes does not occur and the urease enzyme would be inactive. Therefore, a decrease in pH would be necessary to restart the cycling complexation process. Moreover, the system requires the solution to be in the active range of the enzyme. To reduce this well-known issue, the medium would need to be restored to the initial pH.

Moreover, the timing of supplemental urea addition may be significant as well. Supplemental urea would enable further substrate conversion to ammonia if added during the pH evolution. However, the noted chaotropic nature of urea may prevent stomatocyte association; the increased urea concentration alongside the initial addition would provide an ulterior energy barrier for the complex formation to occur, resulting in lower cluster size achieved. If additional urea is added after the first substrate addition is consumed, the chaotropic salt stabilization would be decreased compared to the first scenario. Complex formation and clustering would occur more readily. However, much like dissipative out-of-equilibrium platforms, waste generation represents one of the limiting factors of the system at hand and often reduces output performances in the cyclic repetition of the platforms.^9,18,22,24,40–43^ Therefore, the medium would need to be refreshed to the initial pH, buffer contents, and concentrations.

Elucidating the clustering parameters further and the influence of fluctuating fuel/product concentration gradients are critical in successfully establishing different colloidal communication models, thus translating artificial systems into more biologically relevant and accurate analogs. Lastly, the inclusion of relevant surface moieties can enable the determination of signal diffusion and reception parameters.

## 4. Conclusions

Within this work, we have successfully developed a baseline colloidal system capable of stigmergy-based assembly and disassembly. Uniformly disperse colloidal suspension with encapsulated catalytic functions can reproducibly be obtained and be modified with orthogonal click reactions. Here, surface complementary structures can dynamically cluster at physiological conditions due to the reduced electrical double layer-induced repulsions of the colloids and the complex formation between Ni^2+^–NTA– and His_2_–stomatocytes. Clusters up to twice the diameter of the individual stomatocytes can be reversibly formed by enzymatic action, and diffusion regimes of the catalytic stomatocytes can be altered depending on the extent of interparticle interactions. Given the asymmetry of the stomatocyte morphology, future work should be focused on increasing the enzymatic activity to impart super-diffusive motility regimes. The improvement of the enzymatic motor function coupled with the stigmergic components of this platform may enable higher order functions in stomatocytes, namely clustered collective motion. Overall, the proposed study advances the field of synthetic biology and lays the foundation for a bottom-up development of more elegant and efficient methods for interparticle interactions and complex systems.^44^

## Author contributions

A. D. F., L. K. E. A. A. and T. P. P. designed the experiments and analyzed the experimental results. A. D. F., M. M. E. T., Y. L., and M. C. performed the experiments in this study. J. C. M. H., L. K. E. A. A. and T. P. P. supervised the project. J. C. M. H., L. K. E. A. A., T. P. P., and L. A. provided feedback on the results. A. D. F. wrote the manuscript with the input of all the authors listed.

## Data availability

The data presented in the documents is available upon reasonable request.

## Conflicts of interest

Authors declare no conflict of interest.

## Supplementary Material

TB-012-D4TB01320D-s001
