# Supplementary material for: Enzymatically-induced dynamic assemblies from surface functional stomatocyte nanoreactors
Source: J Mater Chem B. 2024 Oct 7;12(44):11389–401. doi: 10.1039/d4tb01320d (PMC11469296; doi:10.1039/d4tb01320d)
Supplement: TB-012-D4TB01320D-s001 [file TB-012-D4TB01320D-s001.pdf]

Supplementary Information (SI) for Journal of Materials Chemistry B.
This journal is © The Royal Society of Chemistry 2024

ESI - Enzymatically-induced, dynamic Assemblies in Surface Functional Stomatocyte Nanoreactors

NMR spectra of the azide functional macroinitiator, synthesized methoxy- and azide-terminated poly(ethylene glycol)-*b*-poly(styrene) block copolymers

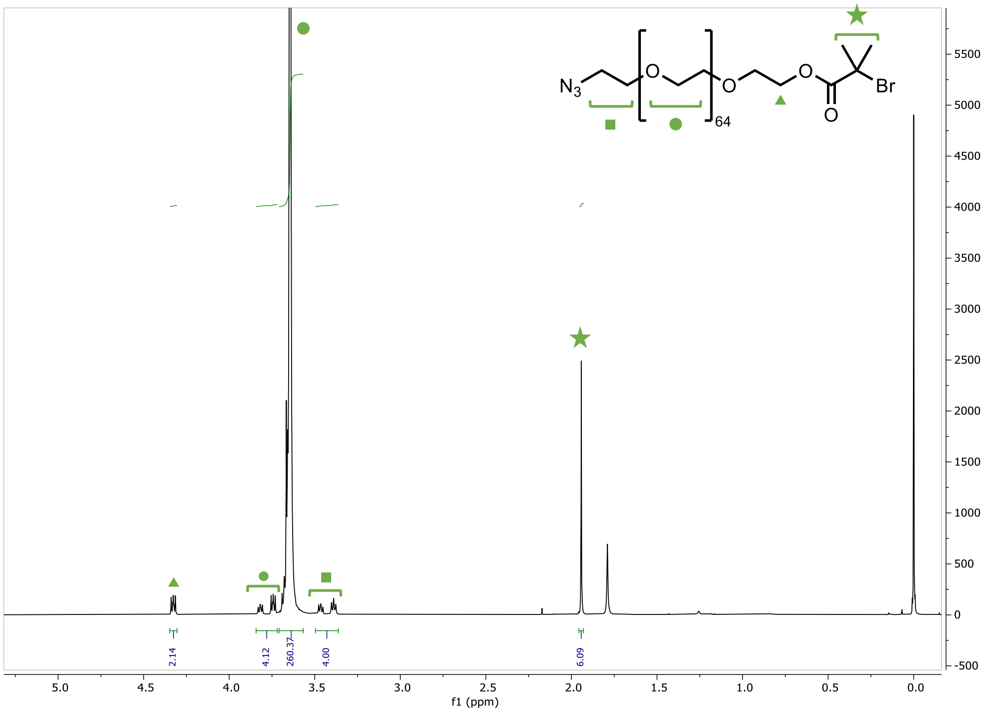

SI Figure. 1 Structure ^{1}H-NMR spectrum and associated peak assignments of the azide-terminated macromolecular initiator.

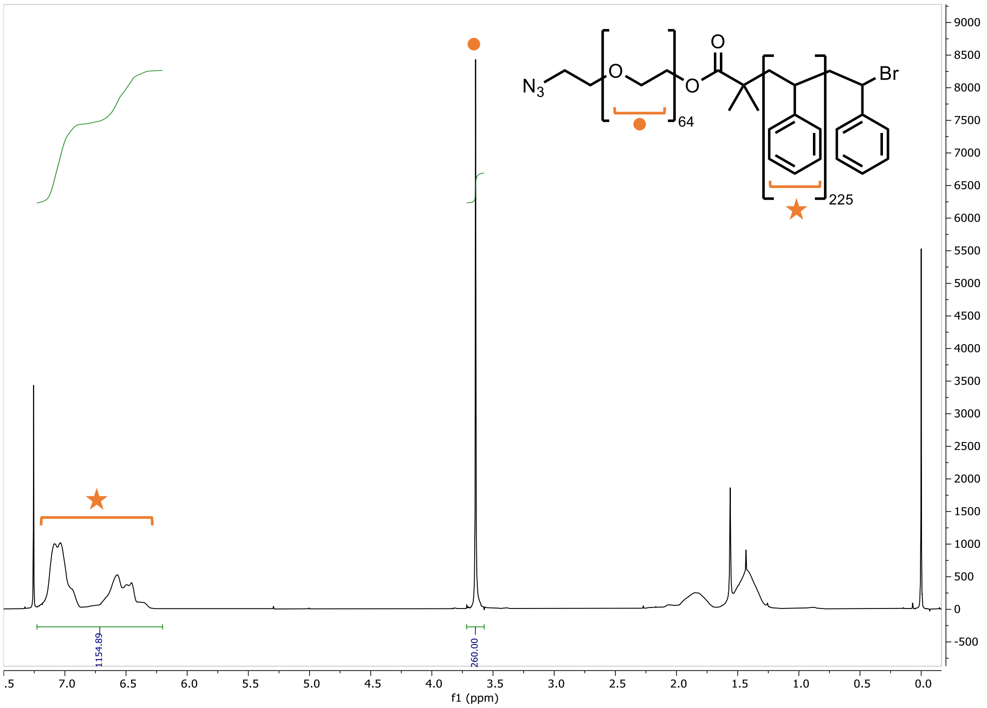

SI Figure. 2 Structure and ^{1}H-NMR spectrum and associated peak assignments of the polymerized azide-terminated polymer.

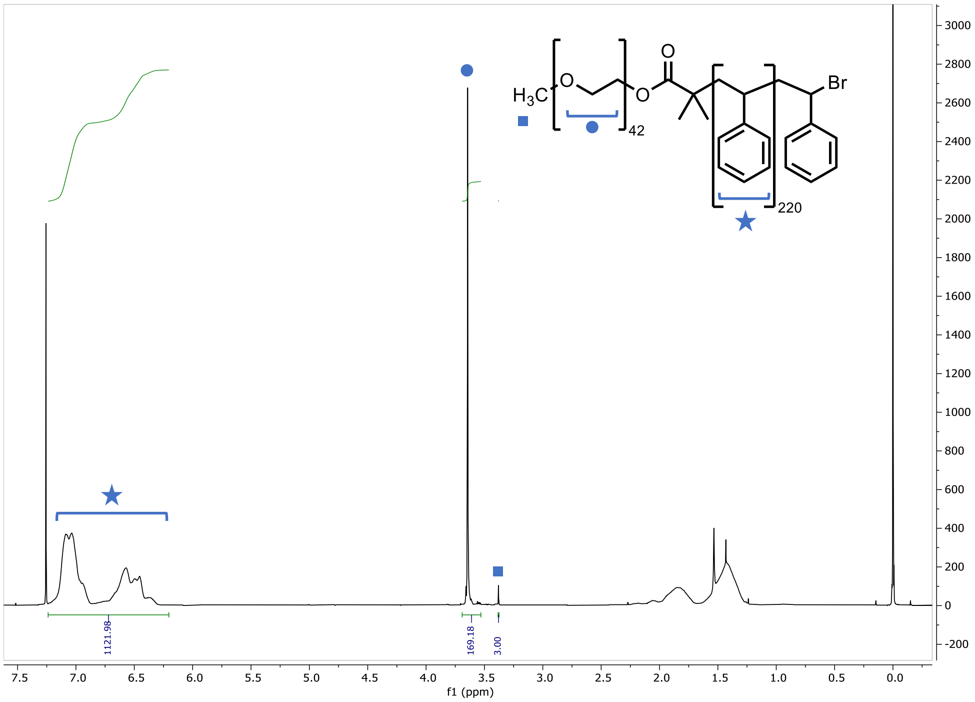

SI Figure. 3 Structure and ^{1}H-NMR spectrum and associated peak assignments of the polymerized methoxy-terminated polymer.

GPC Chromatograms of synthesized polymers and macroinitiators

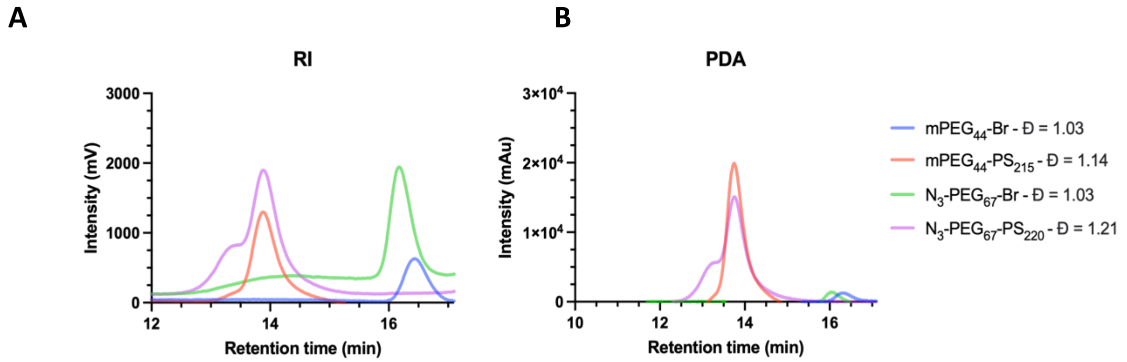

SI Figure. 4 GPC Chromatograms of synthesized polymers. A) Refractive index and B) UV trace chromatograms and dispersity indices of azide- and methoxy-terminated macroinitiators and polymerized diblocks.

Mass spectra of DBCO-His$_2$

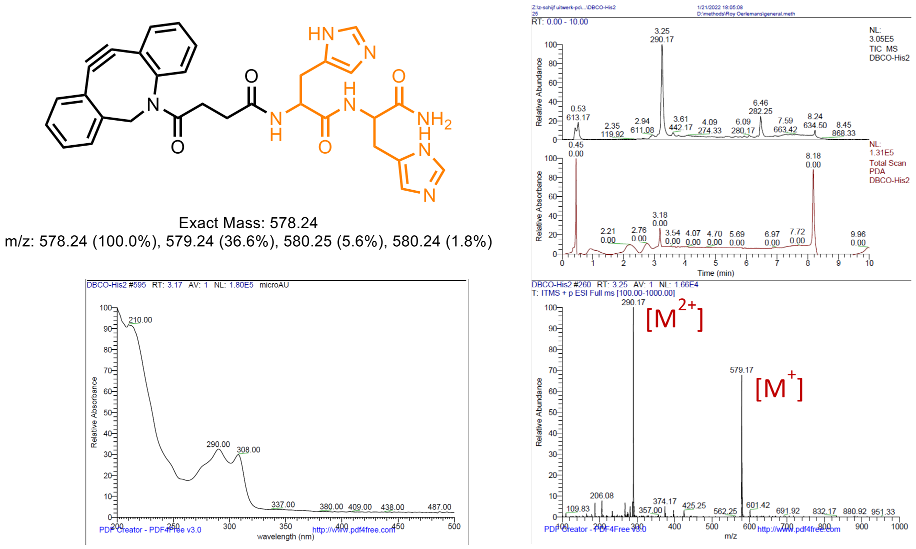

SI Figure. 5 Structure, Mass, and UV spectra of SPPS synthesized DBCO-His$_2$. A) Structures, exact mass, and mass over charge prediction (ChemDraw) of DBCO-His$_2$. B) Liquid Chromatography Mass Spectrometry chromatographic trace of DBCO-His$_2$. C) UV absorption spectrum of DBCO-His$_2$. D) Mass spectrum of DBCO-His$_2$ labeled with with molecular ion peaks.

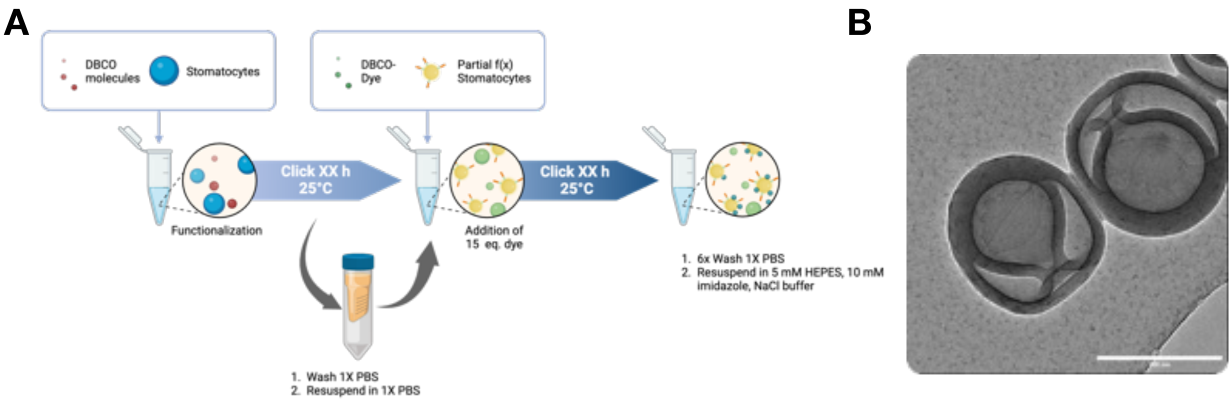

SI Figure. 6 A) Graphical overview of the two-step surface functionalization of stomatocytes by copper-free click reactions. B) Sample Cryo-TEM micrograph focus of closed neck polymersome stomatocytes clicked with DBCO-AZ405 (scale bar = 300 nm).

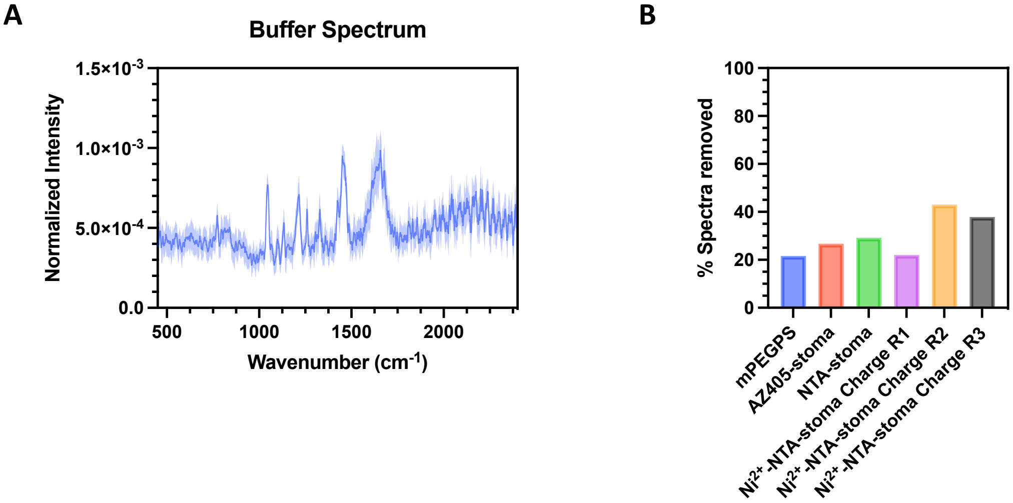

SI Figure. 7 A) Raman spectrum of storage buffer background. Spectrum shown as mean ± s.d. (n=20) after ultrapure water subtraction. Normalized to the area under the curve. B) Percent of spectra removed during pre-processing for each sample due to particle aggregates/clusters.

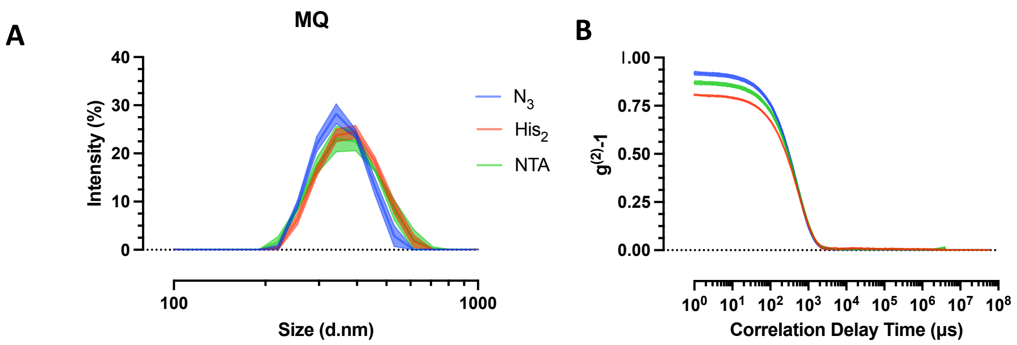

SI Figure. 8 Dynamic Light Scattering data of assembled stomatocytes and surface functional stomatocytes A) Intensity % of colloidal diameters recorded. B) Correlation coefficient of analyzed stomatocytes.

A

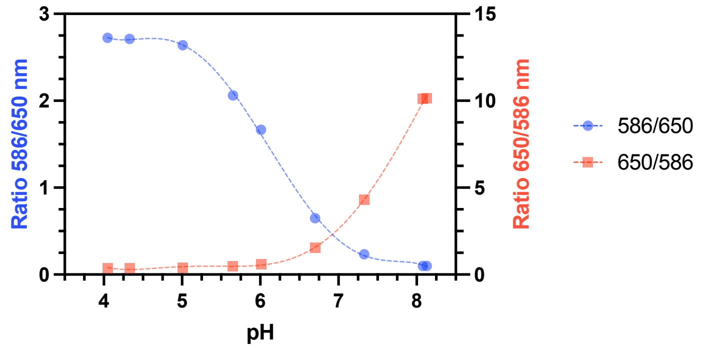

B

	586/650	650/586
**Fifth order polynomial**		
**Best-fit values**		
**B0**	247.7	445.4
**B1**	-222.3	-400.7
**B2**	78.81	141.8
**B3**	-13.62	-24.59
**B4**	1.144	2.083
**B5**	-0.03751	-0.06858
**95% CI (profile likelihood)**		
**B0**	6.377 to 489.1	-567.8 to 1459
**B1**	-433.3 to -11.19	-1287 to 485.3
**B2**	6.017 to 151.6	-163.8 to 447.3
**B3**	-25.99 to -1.239	-76.54 to 27.36
**B4**	0.1066 to 2.182	-2.273 to 6.439
**B5**	-0.07185 to -0.003174	-0.2127 to 0.07555
**Goodness of Fit**		
**Degrees of Freedom**	3	3
**R squared**	0.9995	0.9993
**Sum of Squares**	0.005676	0.1000
**Sy.x**	0.04350	0.1826
**Number of points**		
**# of X values**	9	9
**# Y values analyzed**	9	9

SI Figure. 9 Calibration curve of the ratiometric dye C-SNARF 4F A) C-SNARF 4F emission peaks ratios in 5 mM HEPES, 10 mM NaCl, Imidazole. B) Fifth order polynomial fit of the ratiometric dye.

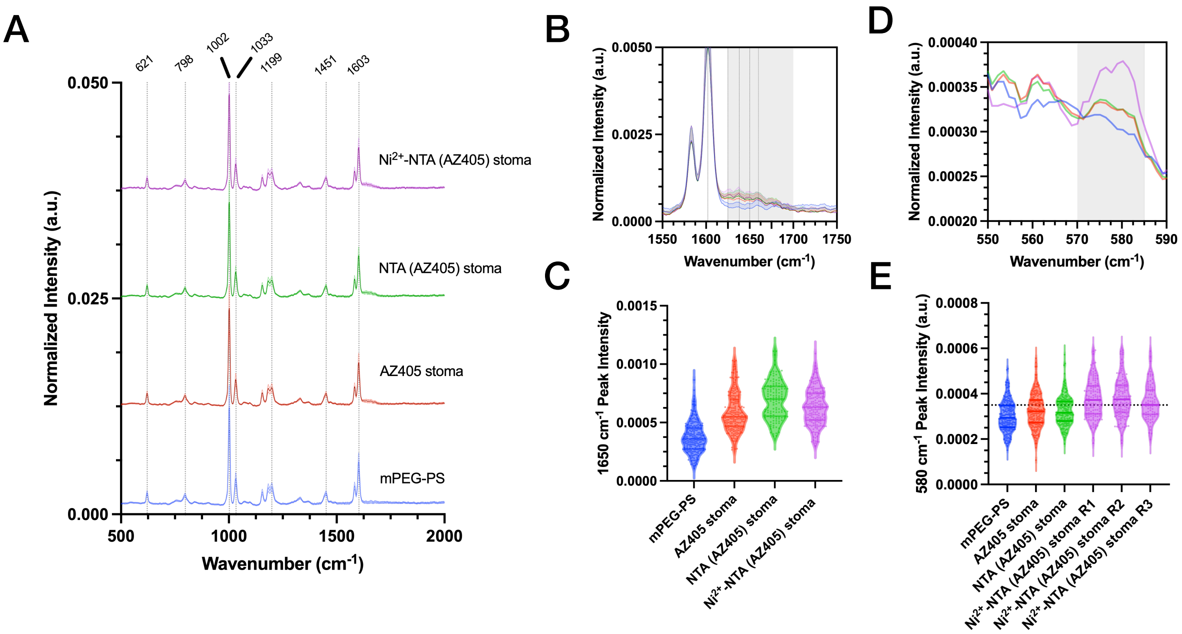

SI Figure. 10 SPARTA analysis of stomatocytes. A) Offset mean single particle Raman spectra of methoxy-terminated, dye-functionalized, NTA and dye functional, and nickel charged PEG-PS stomatocytes with primary peak assignments (mean ± s.d., n>180 particles per sample). B) Mean Raman spectra across each single particle within a sample, cropped to the aromatic double-bonded region (mean ± s.d., n>180 particles per sample). C) Violin plots summarizing the 1650 cm^{-1} peak intensity across individual particles and between sample types (center line, median; upper and lower line, interquartile range). D) Mean single particle Raman spectra zoomed in on the hypothesized Ni^{2+}-NTA mean signal across different sample types at 570 cm^{-1} (mean ± s.d., n>180 particles per sample). F) Violin plots of the Ni^{2+}-NTA peak at 580 cm^{-1} peak intensity across samples (n=77-227 particles per sample type; center line, median; upper and lower line, interquartile range). Dashed line shows upper quartile of the control sample (mPEG-PS).

Table 1. Primary Peak Assignment of Particle Raman Spectra

Assignment	Wavenumber (cm^{-1})
Ring deformation	621
C-H out of plane deformation	798
Ring breathing	1002
C-H in plane deformation	1033
C-C stretch	1154
C_6H_5-C vibration	1199
C-H deformation	‘r1331
CH_2 scissoring	1451
C=C stretch	1583
Ring stretch	1603

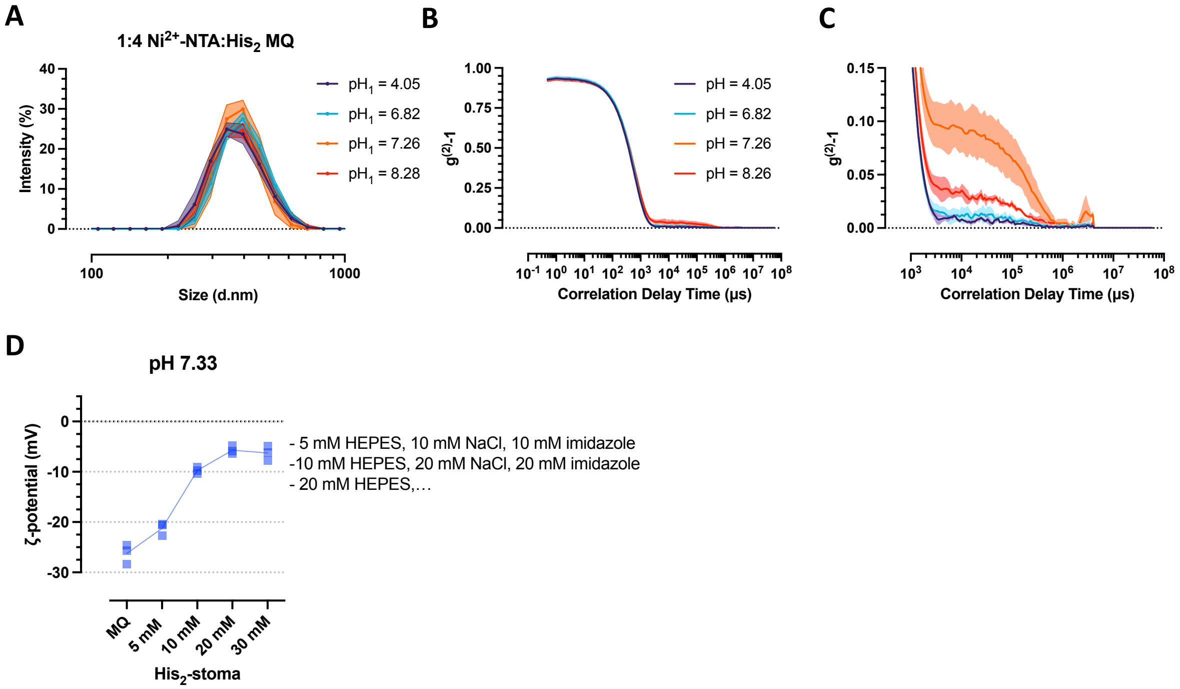

SI Figure. 11 A) Intensity % of stomatocyte cluster hydrodynamic diameters as a function of pH in MQ. B) Correlation function associated with intensity measurements in graph A. C) Correlation function focus at longer delay times indicative of larger colloidal species. D) Zeta-potential measurements of His_2-stomatocytes at pH 7.33 as a function of salt concentrations.

Table 2. Fluorescence intensity output of click reactions in 1X PBS with pH-sensitive ligands

Stomatocyte type	Reaction time (min)	Clicked Surface coverage ratio (ligand+dye/dye)	Coverage with pH sensitive ligand (%)
Nitrilotriacetic acid-stomatocyte	30	0.417 ± 0.0421	58.3 ± 4.21
	60	0.212 ± 0.0145	78.7 ± 1.45
Histidine-stomatocyte	0	0	0
	5	0.389	61.1
	7.5	0.203	79.6
	10	0.140	85.9
	30	0.054	94.5

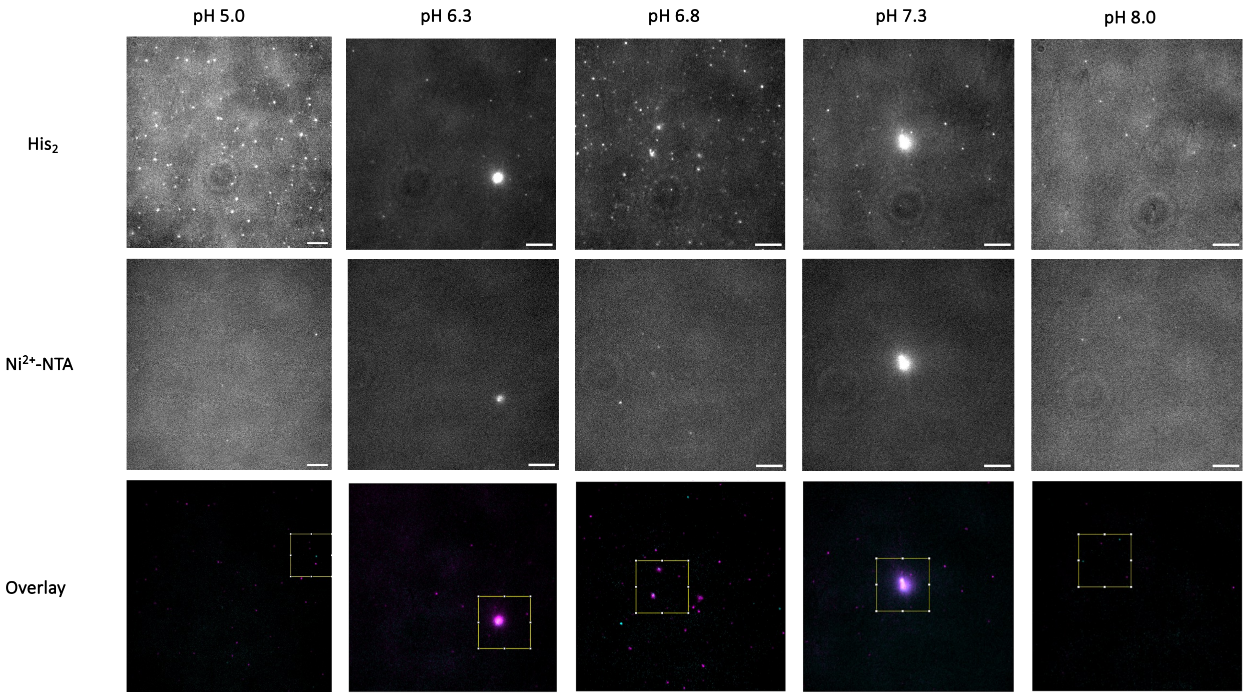

SI Figure. 12 A) Representative fluorescence microscopy images (on glass) of AF488 and AZ647 channels depicting the different particle types at different pHs, overlay and area crops of sedimented Ni^{2+}-NTA- (cyan) and histidine stomatocytes (magenta) populations mixed at a 1 to 1 ratio at different pHs (Scale bar=5 µm).

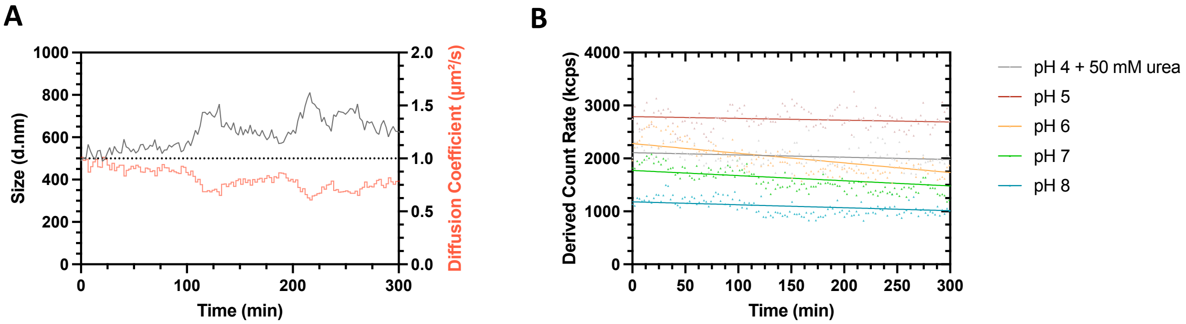

SI Figure. 13 A) Diffusion coefficient and Z_{AVG} of clustering stomatocyte sample recorded over time (50 mM urea). B) Derived count rates and linear fits of clustering stomatocyte mixtures (linear fits included to show trends of derived count rate increases or drops).
